# Psychometric evaluation of the acute care nurses' job satisfaction scale‐revised

**DOI:** 10.1002/nop2.1314

**Published:** 2022-08-21

**Authors:** Yasin M. Yasin, Vahe Kehyayan, Fadi Khraim, Badriya Al‐Lenjawi

**Affiliations:** ^1^ University of Doha for Science and Technology, College of Health Sciences Doha Qatar; ^2^ University of Doha for Science and Technology, College of Business Management Doha Qatar; ^3^ University of Calgary in Qatar Doha Qatar; ^4^ Hamad Medical Corporation Doha Qatar

**Keywords:** acute care, job satisfaction, nurses, psychometric analysis

## Abstract

**Aim:**

The aim of this study was to validate a job satisfaction scale among acute care nurses in the context of Qatar.

**Design:**

Cross‐sectional correlational survey.

**Methods:**

A convenience sampling technique was used to recruit 295 acute care nurses between June 2021–September 2021. Exploratory factor analysis followed by confirmatory factor analysis was used for item reduction and convergent and discriminant validity evaluation. Pearson's correlations were conducted to evaluate the concurrent and convergent validity of the revised scale. Reliability was tested using several internal consistency indicators.

**Results:**

A revised scale was proposed, the Acute Care Nurses Job Satisfaction Scale‐Revised (ACNJSS‐R) scale; it is composed of 13 items loaded on five factors. The composite reliability and the maximal reliability were >.7 for all factors. The study provides empirical support for the validity and reliability of the ACNJSS‐R scale.

## 1 INTRODUCTION

Job satisfaction of acute care nurses is closely associated with several consequences such as absenteeism, job performance, turnover intention, patient satisfaction and patient outcomes (Liu et al., 2016; Lu et al., 2019). Job satisfaction is an essential determinant of acute care nurses' turnover. Various reports in this area of research have highlighted how job dissatisfaction has been associated with higher turnover intention among nurses (De Simone et al., 2018; Yasin et al., 2020a,b). Nurses' turnover has been found to be associated with poor patient outcomes overall (Ghahramanian et al., 2020; Kvist et al., 2014) and negative economical impacts on healthcare systems (Duffield et al., 2014). Identifying acute care nurses' job satisfaction level is critical to improving health service quality and reducing the undesired consequences of nurses' job dissatisfaction (Lu et al., 2019).

To adequately assess job satisfaction and its impact on acute care nurses, valid, reliable and theoretically based scales must be used. After careful review, the Acute Care Nurses Job Satisfaction Scale (ACNJSS) was considered the only scale explicitly designed to assess job satisfaction among acute care nurses (Yasin et al., 2021). Therefore, this study reported further psychometric evaluation of the ACNJSS in the context of Qatar and incorporated additional adjustments based on the factor analysis evaluation.

## 2 BACKGROUND

Nurses are a large and vital segment of any healthcare workforce. A recent report by the World Health Organization (WHO) highlighted the grave concern of policy and decision‐makers over the ongoing shortage of professional nurses that may negatively influence the quality and safety of nursing care (WHO, 2020). Nursing workforce shortages have contributed to several countries not reaching their sustainable development goals, particularly those related to health, healthcare outcomes, patient safety and people‐centred care (WHO, 2020). The increased demand for nursing services is mainly fuelled by the growth in essential nurses' workforce size and expansion of their scope of practice, while the limited supply of nurses is due to factors such as shortage of faculty educators and increased attrition and poor retention of professional nurses (Marć et al., 2019; WHO, 2020). Job satisfaction has been identified as a critical determinant of nurses' retention and their intent to leave the profession (Yasin et al., 2020a). Therefore, proper job satisfaction assessment may mitigate turnover intention and improve the healthcare service quality.

During the past two years, the COVID‐19 pandemic has made the shortage of the nursing workforce more evident. The pandemic has further exacerbated nurses' supply and demand gaps in many countries (Buchan & Catton, 2020). Fear of COVID‐19 has been associated with decreased job satisfaction (Labrague & de los Santos, 2021) and higher turnover intention (Lin et al., 2021). A proper understanding of nurses' job satisfaction and its factors is expected to help identify strategies to improve retention and reduce turnover.

Several factors can affect job satisfaction levels. According to Herzberg's theory, intrinsic (job content) factors such as achievement, recognition, the nature of the work, job‐related responsibility, advancement and growth work as motivators and improve job satisfaction (Herzberg, 1966). In contrast, dissatisfaction from extrinsic (job context) factors such as policies and administration, supervision, salary, interpersonal relations, working conditions and job security lead to job dissatisfaction, but their improvement only has limited long‐term effects on enhancing job satisfaction. Herzberg's theory was used to develop several scales for job satisfaction, such as the ACNJSS (Yasin et al., 2021) and Misener Nurse Practitioner Job Satisfaction Scale (Misener & Cox, 2001).

Job satisfaction is a multifactorial and multidimensional concept. Misener and Cox (2001) defined job satisfaction as a “…multidimensional affective concept that is an interaction of an employee's expectation, values, environment and personal characteristics and it is recognized that satisfiers and dissatisfiers are dynamic and relative to the employee” (p. 93). Liu et al. (2016) concluded that the factors that predict nurses' job satisfaction are demographic, emotional, work character and environment variables. A recent systematic review highlighted the interplay among several factors that influence the job satisfaction of acute care nurses (Yasin et al., 2020b). These factors included poor work conditions, lack of resources, poor staffing ratios, lack of work autonomy and independence, and lack of professional growth among several others that negatively influence nurses' job satisfaction.

Several scales are reportedly used in the literature to assess job satisfaction among nurses. Castaneda and Scanlan (2014) noted that while some scales were developed specifically for nurses, such as the Mueller and McCloskey Satisfaction Scale (Mueller & McCloskey, 1990), others were not specific to nurses, such as the Job Satisfaction Survey designed by Spector (1985). In a systematic review aimed to examine factors associated with acute care nurses' job satisfaction, the authors reported difficulty in comparing results across studies as different scales to assess nurses' job satisfaction were used (Lu et al., 2012). The authors maintained that using different scales essentially resulted in different facets of nurses' job satisfaction being reported across different studies. Spector (1985) pointed out that when a particular scale is developed to assess job satisfaction, those who use the scale in other contexts or professional areas become bound to the facets the scale was designed to assess. For example, Yasin et al. (2021) emphasized that while many scales were used to assess acute nurses' job satisfaction, none were specifically designed to do so among the acute care nurse population.

The ACNJSS was designed to address these concerns particularly as it was specifically developed to assess acute care nurses' job satisfaction (Yasin et al., 2021). Furthermore, as the ACNJSS was developed and used among acute care nurses in Canada, using this scale in another context such as among acute care nurses in Qatar necessitates that the tool be validated. Spector et al. (2015) recommended that measurements used in multinational and cross‐cultural settings be validated and assessed for equivalence. This is particularly important as the majority of acute care nurses in Qatar are expatriates (Al‐Komah et al., 2020). Therefore, this study aimed to validate the ACNJSS in assessing job satisfaction among acute care nurses in the context of Qatar using several validation techniques. In addition, based on the results of this study, a modified version of the ACNJSS was proposed. The revised scale is named as Acute Care Nurses Job Satisfaction Scale‐Revised (ACNJSS‐R).

## 3 THE STUDY

### Design

A cross‐sectional design was used to evaluate the validity and reliability of the ACNJSS‐R in the context of Qatar. The cross‐sectional design is suitable for tool development (Kesmodel, 2018). This design has been used to validate instruments that measure job satisfaction in different contexts (João et al., 2017; Myers et al., 2011).

### Participants

The sample was recruited from four public hospitals in Qatar between June 2021–September 2021. The nursing workforce in these hospitals is one of the most diverse in any healthcare system. This is reflected by the rich diversity in the nationalities of the nurses who come from more than 45 different countries, graduated from varying educational institutions and registered with the regulatory bodies of their respective countries (Hamad Medical Corporation, n.d.). In contrast, the participants in the validation study of the ACNJSS in Ontario, Canada, were Canadian, graduated from Canadian educational institutions and registered with the same regulatory body. Differences in cultural backgrounds influence employee perceptions of workplace environments and contextual factors such as remunerations, promotions and performance recognition (Spector 2015). Furthermore, nurses in Qatar provide healthcare services to a diverse range of patients from different nationalities and cultures who bring their influence to the nurses' work environment.

The required sample size was estimated based on confirmatory factor analysis (CFA) as the primary statistical analysis technique. Myers et al. (2011) have suggested that a minimum sample size of 200 participants has enough power for a confirmatory factor analytic model. The inclusion criteria for this study were Registered Nurses (RNs) working in acute care units for >6 months and having access to a smart device or PC with Internet access to take the web‐based survey. To explore nurse job satisfaction, a minimum amount of working experience in a given context is needed. Six months of minimum experience in the same unit has been recommended as inclusion criteria when investigating job satisfaction among nurses (Kołtuniuk et al., 2021; Yasin et al., 2021). Exclusion criteria included nurses employed in temporary positions or not at the bedside, such as educators, advanced practice nurses or those in managerial positions.

### 3.2 Instruments

The original ACNJSS was developed to measure acute care nurses' job satisfaction levels according to the assumptions of Herzberg's theory (Yasin et al., 2021). Yasin et al. (2021) provided evidence of good psychometric properties. The scale's internal consistency reliability was measured using Cronbach Alpha and ranged between 0.71–0.92 for the scale (Yasin et al., 2021). Content Validity Index was 0.91, and Content Validity Ratios ranged between 0.75–1:00. Exploratory factor analysis (EFA) was used to assess its construct validity, and it suggested a six‐factor model with 31 items (Yasin et al., 2021). The final ACNJSS scale has a Likert scale format with six response options ranging from “very dissatisfied = 1” to “very satisfied = 6.” The identified factors were achievement/job interest/responsibility, hospital policy, quality of supervision, peer support/work condition, growth/advancement and benefits/job security. As mentioned before, most acute care nurses in Qatar are expatriates. The English language is the universal language of communication in Qatar's healthcare system, and therefore, its mastery is a conditional employment requirement. In consequence, all scales used in this study were in English.

The Job Demands in Nursing Scale (JDIN) and Job Resources in Nursing Scale (JRIN) were developed based on the Job Demands‐Resources Model (Penz et al., 2018). The development of both scales went through a three‐phase process, including item selection and development, pilot survey and nationwide survey. The EFA produced a six‐factor structure for both scales where the JDIN scale included 22 items which explained 59% variance in job demand, and the JRIN scale included 24 items which explained 63% of the variance in job resources (Penz et al., 2018). The Cronbach's alpha was 0.84 for the JDIN scale and 0.88 for JRIN scale (Penz et al., 2018). Both scales were used in this study to test for concurrent validity.

Finally, we used a single‐item global job satisfaction scale to further test for convergent validity of the ACNJSS‐R. The global scale is the same one used in the study that produced the ACNJSS (Yasin et al., 2021). The item used to represent global job satisfaction was “On a scale of 1 – 10, with 10 being the highest job satisfaction, how would you describe your overall current job satisfaction?”

### 3.3 Data collection

Data were collected using Qualtrics XM web‐based survey platform between June 2021–September 2021. The survey was sent to a convenience sample of 1,500 nurses working in acute care settings using their work email addresses. As the research team had no access to the participants' email, the nursing research office at the selected hospitals coordinated the survey distribution. Three reminders were sent to the potential participants. The response rate was 21.3% (320 of 1,500 eligible participants). Non‐valid responses were excluded, including unengaged (i.e., linear answers with the same response), outliers and influential responses. After removing incomplete and unengaged responses, 295 complete responses were included in the final analysis.

### 3.4 Data analysis

To evaluate the validity of the proposed ACNJSS‐R, a multi‐step approach was used. Firstly, EFA was done for item reduction and initial construct validity. Then, CFA was performed to evaluate the convergent and discriminant validity. Finally, Pearson's product correlations analyses were conducted to evaluate the concurrent and convergent validity of the revised scale with other related concepts (i.e., job demand, job resources, and global job satisfaction). The composite reliability (CR) and the maximal reliability (MaxR) were used to test the internal consistency reliability. Finally, the data analysis was verified by an independent biostatistician. IBM SPSS version 27 (IBM Corp, 2020) and Amos version 26 (IBM Corp, 2020) were the statistical data analysis programs.

For the EFA, all 31 items in the original ACNJSS in the initial model were included. We used fixed factor numbers to identify the possible factor structure that is similar to the original scale. Maximum likelihood was used as an extraction method because it is the same one adopted for the CFA. Direct Oblimin rotation was chosen due to the expected high correlation between the emerged factors.

For the CFA, chi‐square (*χ* ^2^) test, standardized root mean square residual (SRMR) and root mean square error of the approximation (RMSEA) were the absolute parameters to test for model fit in this study (Kline, 2013). We also reported the comparative fit index (CFI) as the relative parameter model fit (Kline, 2013). The acceptable value for RMSEA is <.06, SRMR is <.05, and CFI is >.95 (Byrne, 2016; Kline, 2013). The value of *χ* ^2^ is preferred to be non‐significant (Kline, 2013). However, due to (*χ* ^2^) sensitivity for the large sample size, this condition cannot be achieved most of the time (Byrne, 2016). Bollen‐Stine bootstrap was performed to validate the final model (Byrne, 2016). Average Variance Extracted (AVE) and Maximum Shared Variance (MSV) were calculated to test for convergent and discriminant validity using the CFA output. The data supporting this study's findings are openly available in figshare repository (Yasin et al., 2022).

### 3.5 Ethical considerations

This study was approved by the University of Calgary Conjoint Health Research Ethics Board (REB21‐0406_MOD2). Furthermore, theregional ethics review of the health institute at Hamad Medical Corporation approvedthe study (MRC‐02‐21‐290).

## 4 RESULTS

### 4.1 Sample characteristics

A total of 295 participants completed the survey with a valid response (i.e., missing values of <10%). The majority of participants were females (71%) and between 31–40 years old (70%). Most participants were married (79%) and had at least a bachelor's degree in nursing (83%). The majority of participants had worked between 11–20 years of total nursing experience (53%). Finally, it is noteworthy to highlight that all the participants in this study were expatriate nurses bringing their respective cultural values to the work environment. See Table 1 for more details.

**TABLE 1:** Sample characteristic (*n* = 295)

Characteristic	Category	Frequency	Percentage
Age (Years)	<30	41	13.9
	Between 31–40	206	69.8
	Between 41–50	40	13.6
	>50	8	2.7
Gender	Male	80	27.1
	Female	209	70.8
	Not answered	6	2.0
Marital status	Single	55	18.6
	Married	232	78.6
	Divorced	1	0.3
	Widowed	1	0.3
	Not answered	6	2.0
Education level	Diploma	16	5.4
	Bachelor	244	82.7
	Graduate	35	11.9
Total experience (Years)	<5	13	4.4
	Between 5–10	100	33.9
	Between 11–20	155	52.5
	>20	27	9.2

### 4.2 EFA

The new factor structure was examined initially for construct validity using EFA. As mentioned above, factor extraction was performed based on fixed factor numbers rather than eigenvalue as the original scale has six factors. The initial factor structure explained 59.7% of the variance in job satisfaction. Further item reduction was performed according to items cross‐loading (≤0.20) and low loading (≤0.40). Two items loading on a single factor were removed from the final model due to low loadings. Consequently, their responding factors were removed, leaving five factors in the final model. This new model of ACNJSS‐R based on EFA was composed of 15‐item compared to the 31‐item in the original ACNJSS. The new model explained 68.9% of the variance. The Kaiser‐Meyer‐Olkin measure of sampling adequacy was 0.92, and Bartlett's test of sphericity was statistically significant (*χ* ^2^ = 3,012.06, df = 105, *p* < .001). See Tables 2 and 3 for more details.

**TABLE 2:** Rotated factor in the pattern matrix

Items	Factor loading
Factor 1: Supervision	
10‐The direct interaction between you and your supervisor	.881
19‐Recognition for your direct superiors	.837
18‐Supervisor support and backup	.821
13‐Supervisor competence	.755
20‐Fairness of assignment distribution	.484
Factor 2: Workplace policy	
15‐The way new policies are implemented	.767
14‐How you are informed about new policies	.736
29‐Opportunity to develop and implement ideas	.553
Factor 3: Growth and advancement	
8‐Opportunity to seek advanced education	.756
11‐Opportunity for professional growth	.627
17‐Opportunity for promotion within the organization	.442
Factor 4: Benefits	
22‐Benefits package	.846
3‐Your salary/hourly wage	.687
Factor 5: Work Environment	
5‐Physical working conditions (lights, noise, cleanliness, heating, ventilation)	.637
28‐Availability of resources and supplies	.615

**TABLE 3:** Correlations among the evolved factors

Factor	Interaction (correlation)				
	1	2	3	4	5
1‐Supervision	1.000				
2‐Workplace policy	.575	1.000			
3‐Growth and advancement	.507	.484	1.000		
4‐Benefits	.523	.529	.422	1.000	
5‐Work Environment	.285	.456	.219	.355	1.000

### 4.3 CFA validity and reliability testing

**FIGURE 1:**
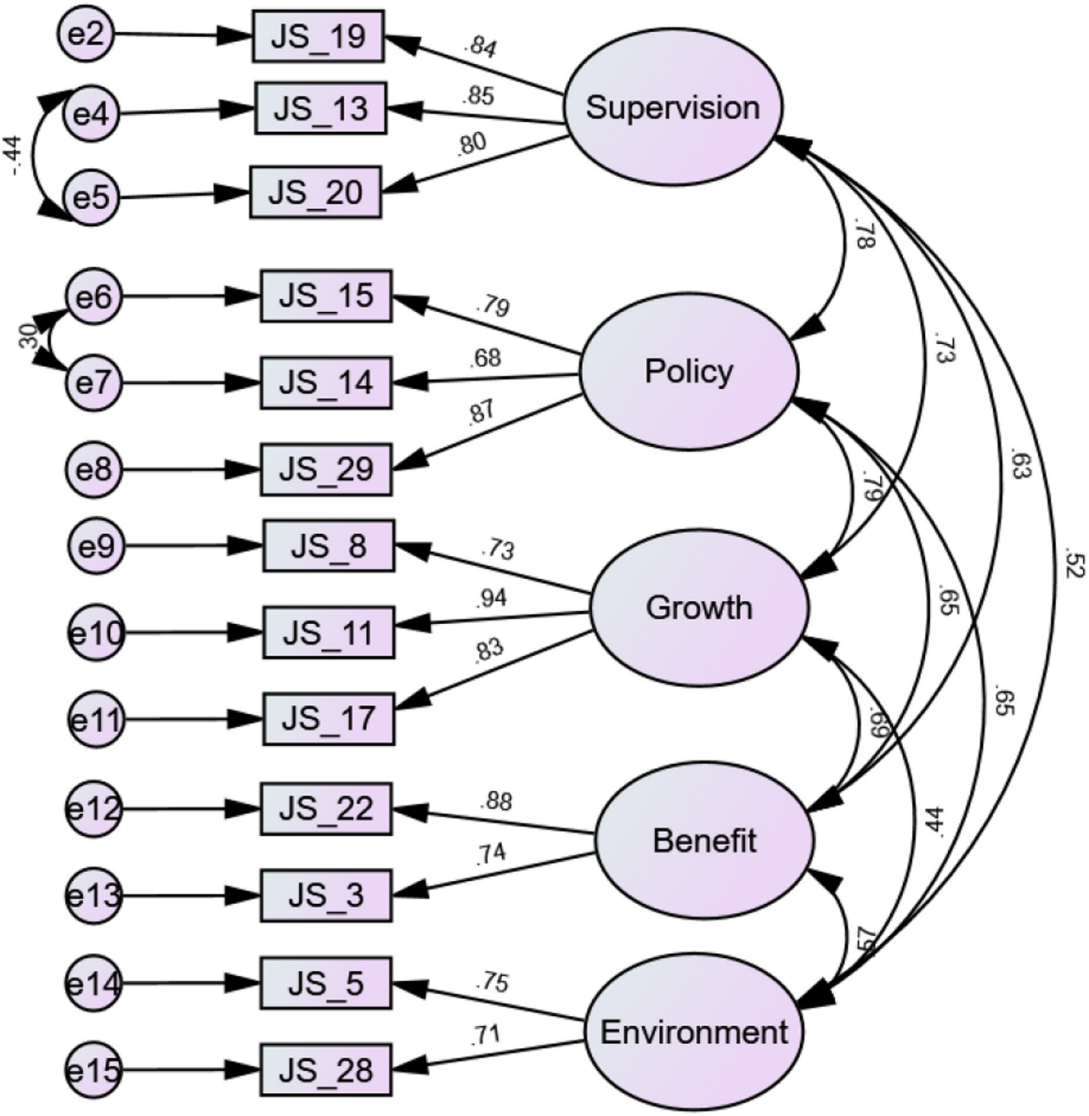
The factor structure of the final ACNJSS‐II with the standardized loadings

The five‐factor solution was tested using CFA with maximum likelihood as an estimation technique. Post hoc analysis and model re‐specification were performed based on the initial results and modification indices; cross‐loading between error terms was allowed in order to improve the model fit if theoretically sound. Finally, two items from the supervision factor were also removed (see Table 4 for the goodness of fit index values). The standardized item loadings for the final model are presented in Figure 1. It can be seen that all items have substantial standardized loadings. All estimated regression weights were statistically significant, and findings were confirmed through bootstrap. The final factor structure comprises the five‐factor model. The names of the new factors were modified from the original scale to supervision, workplace policy, growth and advancement, benefits and work environment (see Table 5).

**TABLE 4:** Summary of model fit index values

Model	*χ* ^2^ (df)	RMSEA (CI)	SRMR	CFI	∆*χ* ^2^, df (*p*)
Model 1 (original)	203.85 (80)	0.073 (0.6–0.85)	0.043	0.958	
Model 2 (removal of item 10)	157.8 (67)	0.068 (0.054–0.082)	0.040	0.965	64.05, 13 (<.001)
Model 3 (covariance between e6 and e7)	139.727 (66)	0.062 (0.047–0.076)	0.038	0.972	18, 1 (<.001)
Model 4 (covariance between e4 and e5)	125.79 (65)	0.056 (0.041–0.071)	0.036	0.977	13.94, 1 (<.001)
Model 5 (removal of item 18)	87.99 (53)	0.047 (0.029–.0.064)	0.032	0.984	37.8, 12 (<.001)

*Note*: Bollen‐Stine Bootstrap for model 5 was insignificant (*p* > .05).

Abbreviations: df, degree of freedom; CI, 90% confidence intervals.

**TABLE 5:** Final factor structure according to CFA

Items	Factor loading
Factor 1: Supervision	
19‐Recognition for your direct superiors	.845
13‐Supervisor competence	.852
20‐Fairness of assignment distribution	.80
Factor 2: Workplace policy	
15‐The way new policies are implemented	.789
14‐How you are informed about new policies	.679
29‐Opportunity to develop and implement ideas	.873
Factor 3: Growth and advancement	
8‐Opportunity to seek advanced education	.726
11‐Opportunity for professional growth	.939
17‐Opportunity for promotion within the organization	.829
Factor 4: Benefits	
22‐Benefits package	.880
3‐Your salary/hourly wage	.743
Factor 5: Work Environment	
5‐Physical working conditions (lights, noise, cleanliness, heating, ventilation)	.753
28‐Availability of resources and supplies	.715

Master validity tool AMOS plugin was used to evaluate the validity and reliability of the final model (Gaskin et al., 2019). The AVE, MSV and the square root of the AVE were calculated. The AVE was lower than CR and >.5 for all emerged factors. The square root of the AVE was higher than any inter‐factors correlation. It is worth noticing that the square root of the AVE for policy is very close to its correlation with growth. The CR and the MaxR were greater than 0.7 supporting internal consistency reliability. See Table 6 for validity and reliability measures.

**TABLE 6:** Validity and reliability

	CR	MaxR (H)	AVE	MSV	Factors correlation with the AVE square root				
					Supervision	Policy	Growth	Benefit	Environment
Supervision	0.87	0.87	0.69	0.60	**0.83**				
Policy	0.82	0.85	0.61	0.61	0.77*	**0.78**			
Growth	0.87	0.91	0.69	0.61	0.73*	0.78*	**0.83**		
Benefit	0.79	0.82	0.66	0.47	0.63*	0.64*	0.68*	**0.81**	
Environment	0.70	0.70	0.53	0.42	0.52*	0.64*	0.44*	0.57*	**0.73**

* *p* < .001.

### 4.4 Correlation of summated scores with related variables

The mean total score for the final 13‐item ACNJSS‐R scale was 3.93 (SD = 0.98), indicating a medium level of job satisfaction. Pearson's product‐moment correlations were used to test the criterion validity of ACNJSS‐R scores and other related concepts. Participants with higher Job Resources in Nursing (JRIN) tend to perceive higher ACNJSS‐R scale scores (*r* = .68, *p* < .001). Conversely, participants with higher Job Demands in Nursing (JDIN) tend to perceive lower ACNJSS‐R scale scores (*r* = −.52, *p* < .001). Furthermore, the correlation between ACNJSS‐R and the global single job satisfaction question showed a strong positive relationship (*r* = .75, *p* < .0001).

## 5 DISCUSSION

The aim of this study was to validate the ACNJSS in assessing job satisfaction among acute care nurses in the context of Qatar using several validation techniques. In addition, based on the results of this study, a modified version of the ACNJSS was proposed. The ACNJSS‐R is a short, self‐administered questionnaire composed of 13 items with five factors: supervision, workplace policy, growth and advancement, benefits and work environment. Its validity and reliability were evaluated using EFA, CFA and correlation analysis. The possible range for the mean score of the ACNJSS‐R is 1 to 6 and maybe interpreted as low (1–2), medium (2.1–4) and high job satisfaction (4.1–6).

### 5.1 Psychometric properties of the ACNJSS‐R

The study findings provided empirical evidence of the ACNJSS‐R validity. The EFA model indicated acceptable convergent validity as all items loading were ≥.40. There were no strong cross‐loadings, and the factor correlation matrix had no value >.7, which supports evidence for discriminant validity. Similarly, all items had high loadings on their assigned factors, with covariance being <.8 in the final CFA model. Further demonstration of convergent validity was evidenced by AVE being >.5 and lower than CR. Discriminant validity was achieved as follows: (a) the AVE was greater than MSV and (b) the square root of the AVE was higher than any inter‐factors correlation (Gaskin et al., 2019).

Another evidence of the scale validity was drawn by testing the correlation between the ACNJSS‐R mean score with related concepts to establish concurrent validity. As expected, there was a statistically significant positive correlation between job satisfaction as measured by the ACNJSS‐R scale and job resources as measured by the JRIN scale. Furthermore, there was a negative correlation between job satisfaction as measured by ACNJSS‐R scale and job demand as measured by the JDIN scale. These findings are consistent with previous studies (McVicar, 2016; Penz et al., 2018). The correlation with an established measure of related concepts is a good example of concurrent validity (Frey, 2018). Finally, the strong correlation between the global job satisfaction score and the ACNJSS‐R mean score is another expression of convergent validity.

Internal consistency reliability was measured in this study using CR and MaxR. The CR and MaxR are often advocated as alternatives to Cronbach's alpha due to the usual violation of the tau‐equivalency assumption (Peterson & Kim, 2013; Sideridis et al., 2018). The findings showed that the ACNJSS‐R demonstrated excellent internal consistency reliability. The CR and the MaxR replaced Cronbach's alpha coefficient in CFA analysis, and values >.7 are considered acceptable (Sharif Nia et al., 2019).

### 5.2 Factor structure

Although the original scale was composed of six factors, the ACNJSS‐R has five factors extracted based on factor analysis. The achievement/job interest/responsibility factor was excluded from the revised scale. According to Herzberg's theory, this factor represents intrinsic factors (Herzberg, 1966). One possible explanation for excluding this factor is that all participants in this study were expatriate nurses who tended to favour extrinsic factors, such as good benefits and better supervision to prevent job dissatisfaction (Yasin et al., 2017). The findings are consistent with other studies in a similar context where expatriate nurses reported job dissatisfaction from extrinsic factors (Alanazi & Yates, 2022). In fact, benefits and workload were the most statistically significant predictors of expatriate nurses' turnover (Alreshidi et al., 2021).

The first factor was supervision, and it is composed of three items reflecting supervisor recognition, competence and fairness. Supervision and leadership were frequently reported in several job satisfaction scales (Lu et al., 2012). Supervisor support (Akinwale & George, 2020; Mazumder et al., 2016), supervisor fairness (Qureshi et al., 2018) and perception of supervisor competence (Feather et al., 2015) were strongly associated with nurses' job satisfaction.

The second factor was workplace policy, with three items reflecting how the new policies in the organization were developed, communicated and implemented. Pandey and Asthana (2017) reported that organizational policy and strategy contributed to employee job satisfaction. A literature review by Hayes et al. (2010) reported organizational policy as a potential influencer on job satisfaction among acute care nurses.

The third factor was growth and advancement, with three items representing advancement, promotion and advanced education. According to the two‐factor theory, growth and advancement are intrinsic job factors that motivate employees and enhance job satisfaction (Herzberg, 1966). In addition, advancement and promotion have been shown to be driving predictors of nurses' job satisfaction (Akinwale & George, 2020).

The fourth factor was benefits, with two items representing benefit and salary. Several studies have shown the association between benefits and salary and job satisfaction (Akinwale & George, 2020; Atefi et al., 2014; Pandey & Asthana, 2017). Furthermore, Artz (2010) found that fringe benefits significantly contribute to employees' job satisfaction.

The final factor was labelled as work environment, with two items referring to nurses' physical working environment and the availability of resources. Albashayreh et al. (2019) indicated that the work environment was responsible for the greatest variation in nurses' job satisfaction. This finding is consistent with two systematic reviews that concluded that the work environment is one of the most reported extrinsic factors affecting acute care nurses' job satisfaction (Lu et al., 2012; Yasin et al., 2020b).

### 5.3 Strengths and limitations

This study had several strengths. Firstly, we had a relatively large sample of 295 nurses working in acute care settings. Secondly, we used several indicators to evaluate the validity of the revised scale. Furthermore, reliability was tested using several internal consistency indicators. This study also had a few limitations worth mentioning. Firstly, its cross‐sectional design and convenience sampling limit the generalization of its finding to other healthcare settings. Second, our response rate was 21% which is lower than what could possibly be expected with paper‐based surveys (Nulty, 2008).

### 5.4 Implications

Senior managers in healthcare organizations could use the ACNJSS‐R as a guide to modify organizational policies in terms of job satisfaction promotion, healthy work environment achievement and reduction of undesired consequences of job dissatisfaction. Furthermore, identifying the level of job satisfaction may help reduce nurses' turnover and limit nursing shortages, especially in times of need, such as the COVID‐19 pandemic. Staff turnover has substantial economic impact on healthcare organizations and patient care outcomes. It is of utmost importance for nurse managers to closely monitor their staff's job satisfaction regularly and periodically. Such monitoring would inform the development of strategies to target areas of dissatisfaction and introduce measures to improve job satisfaction, which would improve the quality of nursing services and patient outcomes. A critical implication to senior management is addressing job demands on nurses and the availability of job resources to them. Higher job resources and lower job demands have the potential of improving job satisfaction among nurses.

Future studies may aim to test the validity and reliability of the ACNJSS‐R in different contexts. Furthermore, future studies may use outcomes of job satisfaction to test for predictive validity. The researchers may use the ACNJSS‐R to measure the job satisfaction of acute care nurses in future studies.

## 6 CONCLUSION

In conclusion, our study has validated the ACNJSS‐R in the Qatari context. The revised scale is composed of 13‐item structured into five factors: supervision, workplace policy, growth and advancement, benefits and work environment. The ACNJSS‐R demonstrated acceptable psychometric properties and can be used to measure job satisfaction among acute care nurses in future studies, occupational health activities and guide modification of healthy work environment policies. In future studies, the use of the ACNJSS‐R scale is recommended.

## AUTHOR CONTRIBUTIONS

The first author conceived of the presented idea and carried out the data analysis and wrote the data analysis section. The second and third authors critically reviewed the manuscript and wrote the background and discussion sections. The fourth author arranged for data collection, responded to ethics board questions and contributed to the interpretation of the results. All authors discussed the results and contributed equally to the final manuscript.

## Data Availability

The data supporting this study’s findings are openly available in figshare repository https://doi.org/10.6084/m9.figshare.19064066.v1
